# Immune Responses to Inhalant Allergens

**DOI:** 10.1097/WOX.0b013e3181788324

**Published:** 2008-06-15

**Authors:** Wayne R Thomas, Belinda J Hales

**Affiliations:** 1 Centre for Child Health Research, University of Western Australia, Telethon Institute for Health Research, Western Australia

**Keywords:** allergens, B cells, T cells, IgG antibody, IgE antibody, immunoregulation

## Abstract

This overview describes the nature of the immune responses induced by the inhalation of allergens. There is a dichotomy in that B cells have multiple mechanisms that limit the amount of immunoglobulin E (IgE) antibody production, whereas T-cell responses are large even in nonallergic subjects. With the possible exception of responses to cat allergen, however, T cells from nonallergic subjects have limited effector function of helping IgG antibody, and in house-dust mite allergy, this declines with age. Regulation by interleukin 10 (IL-10)-producing cells and CD25^+ ^T-regulatory cells has been proposed, but critically, there is limited evidence for this, and many studies show the highest IL-10 production by cells from allergic subjects. Recent studies have shown the importance of nonlymphoid chemokines thymic stromal lymphopoietin and IL-27, so studying responses in situ is critical. Most sources of allergens have 1 or 2 dominant allergens, and for house-dust mite, it has been shown that people have a predictable responsiveness to high-, mid-and poor-IgE-binding proteins regardless of the total size of their response. This allergen hierarchy can be used to design improved allergen preparations and to investigate how antiallergen responses are regulated.

## Immunoglobulin E Responses to Allergens

A hallmark of immediate hypersensitivity is the ability of mast cells and basophils armed with small amounts of immunoglobulin E (IgE) antibody to induce powerful inflammatory reactions. Immunoglobulin E is indeed produced in lower amounts than the more common IgG isotypes. The serum immunoglobulin levels, which vary with atopic status, are in the region of 5 to 500 ng/mL compared with 0.5 and 10 mg/mL of IgG4 and IgG1, respectively. There are few IgE-producing cells, and IgE has a short half-life. Recent studies show that the IgE antibody titers in humans correlate very well with the presence of IgE producing early plasma cells in the blood, [1] suggesting that the IgE response is predominantly caused by short-lived cells, and this is compatible with the diminution of IgE antipollen titers found between pollen seasons. There are at least 2 control points at the molecular level. The rate of translocation of the *Vh *genes to the *0 *gene in class switching is intrinsically weak compared with the switching for gamma subclasses, [2] and RNA transcripts for membrane IgE are unstable compared with the IgG counterparts [3]. At the cellular level, it has been shown in mice that B cells with surface IgE are exceptional because they were largely found outside germinal centers [4]. They arise from a pre-IgE phase, where somatic mutation and affinity maturation take place in IgG1^+ ^cells, and a post-IgE-switching phase, in which IgE cells differentiate swiftly into plasma cells. This leaves a deficiency in IgE-bearing memory cells that would maintain long-lived responses. Studies on the immunoglobulin heavy chain repertoire expressed by antiallergen IgE antibodies show that there are no restrictions with respect to the macroelements of repertoire diversification such as the selection of *Vh *gene families or VDJ recombinations, [5] but that the responses show a lower repertoire with respect to the number of different *Vh *genes. Selection from continued exposure to low doses of antigen or limitations of antigen binding imposed by the unique structure of IgE could account for this. For the last possibility, it has been proposed that steric hindrance of antigen binding might occur because IgE has a bent structure that places the Vh region in close proximity to the C-terminal of the constant region [6].

Consistent with tight control mechanisms, it has been repeatedly demonstrated that antiallergen antibodies constitute a large portion of the IgE immunoglobulin [7-9]. The size of the average IgE antibody response varies with the nature of the allergen source. The serum concentration of IgE antibody binding to the dominant allergens of birch, [10] grass, [11] and mite[12,13] are about 50 ng/mL, whereas Amb a 1 has been reported as 20 ng/mL, [14] and the cockroach Bla g 2 and 5 have a combined average of 10 ng/mL [15]. Many people have low levels of IgE to cat, [16,17] so the average anti-Fel d 1 levels are about 4 ng/mL. Some people, however, have very high levels, with more than 100 ng/mL [18]. The Can f 1 dog allergen has comparable IgE titers to Fel d 1, but only a small sample of subjects has been examined [16]. Immunoglobulin E antibodies to the mouse allergen Mus m 1 are also present in small quantities, with an average of about 1 ng/mL in subjects that develop disease after domestic and industrial exposure [19]. There is a possibility that lower evolutionary divergence contributes to lower responses to mammalian allergens, but given that Fel d 1 only has 20% identity with the human uteroglobin homologue, other factors such as the aerodynamics of the allergen may be involved.

The engagement of IgE with the high-affinity FcεR1 in the absence of allergen increases the expression of the receptor on the surface of basophils, mast cells, and dendritic cells, so that the IgE levels, and thus the type of allergen-inducing sensitization, can directly affect the allergic response. It was thought that this occurred by inducing the synthesis of FcεR1, but it now seems to occur via a membrane-stabilizing process [20].

The development of allergic symptoms is related to the titers of antiallergen IgE antibody, but even children with the highest titers only have a 60% probability of disease as shown for wheeze in asthma, and although the probability declined with reduced titers, it was still elevated at the low level of 4.4 ng/mL [21]. The classification of the disease must also be considered. In house dust mite (HDM) allergy, for example, only children with the highest IgE antibody titers have persistent asthma, [22] a very severe form of the disease. Exacerbation of intermittent asthma for example by infection is a more frequent health problem, producing 75% of hospital admissions for asthma in children, [23] and many such children with mite-allergy have quite low titers, less than 10 ng/mL [13].

## T-Cell Responses

In contrast to IgE antibody, the T-cell responses to allergens are large. The precursor frequencies of T cells responding to allergen extracts have been reported for pollen[24] and HDM allergy [25,26]. For mites, the frequencies of T-cell precursors in the peripheral blood were in the range of 0.05% to 0.1% for allergic subjects and 0.01% to 0.02% for nonallergic subjects. Subjects allergic to birch and grass pollen had precursor frequencies with an average of 0.56% for symptomatic subjects and 0.12% for nonallergic nonatopic people. Assing et al [24] also determined the precursor frequencies in people who had skin test reactions but were asymptomatic, finding a frequency of 0.13%, being the same as skin test-negative controls. The asymptomatic skin test-positive people, however, had low levels of IgE antibody and skin test reactivity compared with the symptomatic subjects, so this does not provide information on why some subjects with high levels of sensitization have few symptoms. For comparison with allergens, frequencies in the region of 0.001% for KLH[27] Leishmania[28] and hepatitis B[29] have been found before immunization and rise to about 0.02% after vaccinations. Thus, even nonallergic subjects show a large degree of expansion. The allergen-responsive T cells of allergic subjects are mainly in the memory CD45RO^+ ^population[24,25] compartment, whereas allergen-responsive cells are found in both the CD45RO^+ ^and CD45RA^+ ^compartments of nonallergic subjects [25]. The induction of the Th2 transcription factor GATA-3 by HDM extract has also been shown to be stronger in CD45RO^+ ^cells [30].

Although most of the T-cell responses in allergy have been studied with extracts, the use of purified allergens is more informative because the stimulating specificities and the concentration used is known and can be accounted for. The T-cell responses to the dominant allergens from grass pollen, [31,32] birch, [33] weeds, [34,35] mite, [36] and cat[37] have been studied. The proliferative responses induced in the peripheral blood mononuclear cells (PBMCs) from allergic people are generally better than those of cells from nonallergic people, although there is considerable overlap [36]. The induction of the Th2 cytokines interleukin 5 (IL-5) and IL-13 can be readily detected, [38-40] although IL-4 is typically produced in low amounts. Contrary to early observations with extended T-cell culture, PBMCs from allergic subjects show the same amount of interferon-γ (IFN-γ) release as cells from nonallergic subjects as demonstrated with mite[38-41]and pollen allergens [24,41,42]. Some studies have even shown increased IFN-γ [43-45]. The amount of IFN-γ induced by allergens is very similar to that reported to be induced by Th1-type bacterial antigens and vaccines, [46-48] so they are not trivial responses. It is likely that the early observations of low IFN-γ from allergic subjects were caused by the potent inhibitory activity of IL-4 on Th1 responses in culture. An interesting observation of Assing et al [49] was that IL-2 was released in excess in cultures of PBMCs from symptomatic pollen-sensitized people and could not be detected in nonsymptomatic and skin test-negative people. Not only is IL-2 produced in larger amounts by Th1 cells, but it is also important for the induction of regulatory T cells.

CD4^+ ^cells have been shown to be the predominant source of Th2 cytokines in PBMCs[50] and to predominate in the cellular infiltrates of airways [51]. The Der p 1 allergen has, however, also been shown to stimulate the release of IFN-γ from CD8^+ ^cells although in in vitro cultures, provided that they were supplemented with IL-2 [43]. In a later article, Der p1-specific CD8 cells were detected by isolating T cells with a major histocompatibility complex class I binding peptide tetramer [52].

Studies on cytokine production in situ have shown that the Th2 polarization is best found in the lungs and not the peripheral blood [53]. Thymic stromal lymphopoietin (TSLP) could have a large effect in the target organ. This epithelial cell product, [54] presumably stimulated by tissue damage, is a powerful inducer of Th2 responses, mediating both expansion and polarization while maintaining the central T-cell memory [55]. It is in turn stimulated by Th2 cytokines and TNF-α and shown to be unaffected by IL-10, IFN-γ, and transforming growth factor-β (TGF-β). Recent studies have identified that T-cell receptor-activated T cells express the TSLP receptor, thus providing a marker for the allergy-mediating cells [56]. This would permit a direct action on T cells as well as the previously known effect via the dendritic cells. The IL-27 secreted by macrophages and dendritic cells may have a reciprocal role, having a potent suppressive action on Th2 as well as Th17 responses [57]. The cytokine bias of T cells in vivo can be tracked to some extent by their chemokine receptors. The Th2 cells preferentially express the chemokine receptors CCR3, CCR4, and CCR8 and migrate to their respective ligands, eotaxin (CCL11), monocyte-derived chemokine (MDC) (CCL22) and thymus- and activation-regulated chemokine (TARC) (CCL17). Bronchial lavages of asthmatic subjects show the accumulation of CCR4^+ ^CD4^+ ^cells and the secretions of the corresponding chemokines TARC and MDC [58]. Endobronchial biopsies after allergen challenge revealed that most of the infiltrating T cells expressed CCR4 with some coexpression of CCR8, and the epithelial cells produced MDC and TARC [59,60]. The Th1-type IP-10 chemokine can also be produced in asthma, as shown by its production after segmental lung challenges with ragweed, HDM, and cat extracts [61,62]. Patients who produced late-phase reactions to allergen challenge produce more of both the Th1 and Th2 chemokines [61].

The chemokine receptor bias of allergen-responsive T cells to CCR4 has also been detected in grass pollen allergy [24,63]. The PBMCs of allergic subjects also preferentially produce the corresponding Th2 TARC and MDC compared with cells from nonallergic people [64,65]. Recently, pollen allergen-responsive CCR4 cells have been shown to be increased in the pollen season and to be associated with the increased CD62L CD45RO markers for central memory [66]. Allergen-stimulated cells can also be tracked ex vivo by tetramer-binding cells. A study of CD4^+ ^T cells from atopic dermatitis patients with IgE antibody to the cat allergen Fel d 1 used tetramer binding to show a similar result, with most of the T cells being within the central memory compartment [67]. Their frequency was about 0.01% for cells from atopic dermatitis patients and about 0.002% for cells from nonallergic people. Both of these are also high, considering that only 1 epitope bound to 1 HLA-DR molecule was measured. Cat exposure was not reported.

## Regulatory T-Cell Responses

There are many observations that show that patients undergoing immunotherapy produce more IL-10 and TGF-β, [68] but it is not clear if these regulators have any control over the development of allergic sensitization. There is considerable significance given to the report by Akdis et al, [41] showing that Der p 1 and pollen allergen-stimulated cells from every nonallergic person examined produced more IL-10 than cells from every allergic person examined. Clear-cut results have not been obtained by other studies. A nonsignificant trend for less IL-10 production by cells from symptomatic cat- and pollen-sensitive children has been reported, [69] but most studies have found that IL-10 is produced in higher amounts by cells from allergic subjects. This has been shown for purified HDM allergens[40,45,70] and for cells stimulated with cat and pollen extracts [49,71,72]. A study with pure Fel d 1 found that cells from cat-allergic and nonallergic subjects produced the same amount of IL-10, [37] and the latter included people producing IgG4 antibodies. A recent study of Bet v 1 stimulation in birch pollen allergy showed no difference in the IL-10 release induced in cells from allergic and nonallergic subjects, [42] as previously reported by Bullens et al [73]. Bullens et al [73] also found similar levels of IL-10 induced by Der p 2 stimulation [74]. Increased IL-10 has also been found in bronchial and skin challenge sites of HDM allergic people, as indicated by messenger RNA transcription [75].

Evidence that IL-10 might regulate the responses of cells from healthy individuals has been obtained by experiments where the addition of anti-IL-10 receptor antibodies to cultures of their PBMCs enhanced proliferative responses to Der p 1 [76]. This observation was not reproduced in the study with Bet v 1, [42] although anti-IL-10 did increase the production of IFN-γ and TNF-α but not Th2 cytokines. The possibility of a regulatory role for IL-10 in allergic subjects has been indicated in HDM allergy, where patients who produce more IL-10 had smaller wheal sizes in skin prick tests [45,70]. Opposite to this, however, has been the demonstration that children with early and persistent allergy have more disease and higher Th2 cytokine responses than children with late-onset allergy and that they also have higher IL-10 responses [77].

Suppressive effects of CD4^+^CD25^+ ^T-regulatory cells have been demonstrated on the proliferation responses of PBMCs cultured with cat and pollen allergens. There was, however, no convincing difference in the activity of cells from allergic and nonallergic subjects[78,79] or the inhibition of effector events such as cytokine production. The study of Ling et al [78] did show decreased suppression within the pollen season, but because the T-cell responses were much higher than outside of the season, the effect is difficult to interpret. A study of birch allergy outside of the pollen season found that although nonallergic subjects and patients tended to have reduced IL-10 release from the CD25^+ ^cells, it was possible to detect a suppressive activity on T-cell proliferation for the cells from allergic, but not nonallergic, subjects [42]. Inspection of the data, however, suggests that this was caused by uncharacteristically low proliferative responses of the cells from allergic subjects and a higher proliferation of the CD25^+ ^population itself. The Th2 cytokine responses were not affected by the presence of CD25^+ ^cells. Studies of atopic dermatitis showed that HDM extract stimulated more of the regulatory cell transcription factor FOXP3 from PBMCs from HDM-allergic subjects than PBMCs from nonallergic subjects, [80] and that pollen-allergic children have increased numbers of CD4^+ ^CD25^+ ^cells [81]. Studies of T-regulatory cells are, however, clouded by the knowledge that both CD25 and FOXP3 are induced in activated effector and regulatory T cells, albeit transiently [82]. CD4^+^CD25^+ ^high or bright cells are considered to have high regulatory activity, with few or no effector T cells. However, the CD4^+^CD25^+ ^high subgroup has been shown to contain a mixture of both regulatory and effector T cells, and the percentage of CD4^+^CD25 high T cells correlated negatively with the suppressive capacity of CD4^+^CD25^+ ^T cells [81]. There is an absolute requirement for TGF-β in the development of T-regulatory cells, and the ability of a TGF-β antagonist to increase T-cell proliferative responses to Der p 1 has been reported [76]. Although the experimental studies to date have been inconclusive, an overall role of T-regulatory cells is demonstrated by the increased allergic sensitization in FOXP3-deficient immunodysregulation, polyendocrinopathy, enteropathy, X-linked patients [83].

## IgG Antibody Responses

There is good agreement that IgG antibodies to grass, [84] mite, [13,70,86] ragweed, [85] and birch[87] allergens are predominantly found in sera of people with IgE antibodies. It therefore appears that although nonsensitized people have T-cell responses to these allergens, they do not make antibody responses of significant magnitude. For mites, some studies with mite extracts have reported pan IgG in nonallergic subjects, but this is difficult to measure, and the method of differentiating from nonspecific binding was not given [88]. Not all allergic people produce IgG, with Hales et al [13] finding IgG in 70% of mite-allergic children but only in 40% of adults. Furthermore, only 25% of children admitted to an emergency department had IgG and the titers were low, suggesting a relationship with susceptibility to exacerbation [13]. Jarvis et al [88] reported an association of IgG4 with symptoms in adults, but this analysis only measured the general association of IgG found in sensitized subjects. The titers of antibodies to the major HDM allergens reach about 0.5 μg/mL for IgG4 and 10 μg/mL for IgG1. These titers are similar to the antibody titers found to a *Haemophilus influenzae *P6 protein antigen using the same technique and other antibacterial responses [89,90]. They are however lower than levels that would induce antibody-mediated pneumonitis [91]. Cockroach IgG antibodies are mainly also associated with IgE responses [15]. Grass pollen allergens induce lower IgG antibody titers than mite with mainly IgG4 antibodies at less than 1 μg/mL [92].

Fel d 1 has been reported to induce IgG antibodies in most people exposed to cats[93] possibly because it is present in inhalable air in 50-to 100-fold higher concentrations than mite and pollen allergens [94]. It has been proposed that this tolerizes for IgE while maintaining IgG production [93]. A recent study, however, found that IgG antibodies to cat were 10-fold higher in people with IgE [88]. Mouse allergens have also been reported to induce IgG in nonallergic people in studies of occupational exposure, [95] but data from domestic exposure showed a strong association of IgG antibody with IgE. This may be related to the 50 times lower amounts of Mus m 1 in the air of homes compared with Fel d 1 [95].

## Anti-HDM Responses are Hierarchical

Most of the IgE binding to the common sources of allergen is directed to a small number of dominant allergens. Bet v 1 is a very dominant allergen binding 90% of the IgE antibodies to birch pollen in sera from people in Scandinavia [96]. Ole e 1 has a similar dominance for olive pollen, [97] and the group 1 and 5 allergens from grass pollen allergens collectively bind 80% of the IgE in 95% of sera [11]. Amb a 1 is the dominant allergen for ragweed, binding a range from 25% to 85% of the IgE antibodies to pollen extracts [14]. Immunoglobulin E antibodies to Fel d 1 constitute about 50% of the IgE binding to cat dander extracts, [18] although this may not be the major source of all cat allergens [17]. The combination of the group 1 and 2 HDM allergens binds over 50% of the antimite antibodies [12,13].

To examine the relative binding of the nonmajor mite allergens to the known major Der p 1 and Der p 2 allergens, a panel of 9 allergens was prepared including allergens known to absorb out the IgE binding to nearly all the allergens detectable by IgE immunoblotting of 2-dimensional electrophoresed extracts [98]. The allergens used were either natural allergens or recombinant allergens whose structure had been validated by biochemical function or allergenicity. All the antiallergen antibody measurements were conducted in standardized conditions, so the titers were not influenced by the amount of allergen [13]. There was an excellent correlation of the summated titers of IgE to the different allergens with the values obtained by the Phadebas CAP system with mite extract. Immunoglobulin E antibodies to Der p 1 and 2 made up about 50% of the binding, regardless of the size of the total response or the presence of symptoms. Immunoglobulin E binding to Der p 4, 5, and 7 collectively accounted for 30% of the IgE, and this varied in proportion to the titers to Der p 1 and 2. Binding to Der p 3, 8, 10, and 20 was quantitatively low for nearly all people, although a high frequency of binding was found for some of the allergens. The titers to the midpotency Der p 4, 5, and 7 allergens of 10 ng/mL indicate that they could be important. Comparison with other studies indicates that IgE antibody titers to Der p 6, 9, 13, 16, and 17 will also be very low [99]. Immunoglobulin E binding therefore has a hierarchy that is determined by the allergen. The finding that there is the same hierarchy of response in low and high responders means that different formulations to better represent HDM allergens than extracts can be rationally tested. The IgG antibody response to the allergens shows the same hierarchy as the IgE binding, with the highest titer being found to Der p 1 and 2 followed by Der p 4, 5, and 7. The same mix of IgG1 and the highly Th2-dependent IgG4 antibodies was found. There is at this stage no evidence for a nonallergenic response to poor allergens. A caveat to this study is that allergens of possible importance, namely Der p 11, 14, and 15 have not been studied because they have not been produced or purified in the necessary amounts. The study of the production of mite allergens has shown that poorly allergenic molecules such as mite ferritin, the fatty acid binding protein, tropomyosin, and arginine kinase are produced in larger quantities than the major group 1 and 2 allergens and the midpotency group 4, 5, and 7 allergens [100].

## Conclusions

The IgE antibodies induced by allergens are produced in low quantity, and because several features of the B-cell response seem to contribute to this, the system seems tightly regulated. In contrast, the precursor frequency and the amount of cytokine produced by T cells responding to allergens are high and of a similar magnitude to those found for antimicrobial responses. They tend to be higher for allergic compared with nonallergic people, but even the responses of nonsensitized people are similar to those induced by vaccine antigens. The IgG antibody titers are also higher in allergic people, at least for responses to HDM, pollen, and cockroach and mouse allergens. The titers to pollen are small, but the magnitude of the responses to HDM allergens in children are similar to antimicrobial responses and include the IgG1 isotype found in antimicrobial immunity and the highly Th2-dependent IgG4 isotype. Only about 70% of children however produce IgG, and the responses decline with age. It was also found to be severely curtailed in children with intermittent asthma experiencing acute exacerbations. The mechanisms that regulate the IgG responses and perhaps the direct influence of the presence of IgG could therefore be important for disease. The lack of any antibody in nonallergic subjects indicates an inefficient helper T cell function, although allergic and nonallergic subjects have good cytokine responses, as shown by IFN-γ release. The IFN-γ responses of both allergic and nonallergic people are similar and of a similar size to those found in microbial antigens. It is becoming clear that chemokine responses by nonlymphoid cells, especially TSLP and possibly IL-27, are critical regulators of the allergic response, so that responses studied in extended in vitro cultures are not necessarily very informative. Regulatory responses by IL-10-producing cells and CD25^+ ^T-regulatory cells have been proposed as important modifiers of the responses of nonallergic subjects. It is however important to note that the evidence for this is limited, and most studies show that peripheral blood cells from allergic subjects produce more or similar amounts of IL-10 to the cells from nonallergic subjects. This, however, could be the result of an interplay between cytokines in the in vitro culture systems. At least some people exposed to cats make high IgG4 responses to Fel d 1 without the production of IgE antibody, and this could be the result of a regulatory function. It is however possible that the frequency at which this occurs is lower than previously suspected. For HDM allergy, it has been shown that the IgE and IgG responses induced by the different allergens are hierarchical, with the proportion of the response directed to each allergen being similar for all individuals. This not only has use for designing improved allergen preparations, but also can be used as an investigative tool for determining how the responses are controlled.

## End note

Supported by the National Health and Medical Research Council of Australia.
